# Intraoperative imaging identifies thymoma margins following neoadjuvant chemotherapy

**DOI:** 10.18632/oncotarget.6578

**Published:** 2015-12-12

**Authors:** Jane J. Keating, Sarah Nims, Ollin Venegas, Jack Jiang, David Holt, John C. Kucharczuk, Charuhas Deshpande, Sunil Singhal

**Affiliations:** ^1^ Division of Thoracic Surgery, Department of Surgery, University of Pennsylvania, Perelman School of Medicine, Philadelphia, PA, USA; ^2^ Department of Clinical Studies, University of Pennsylvania School of Veterinary Medicine, Perelman School of Medicine, Philadelphia, PA, USA; ^3^ Department of Pathology, University of Pennsylvania, Perelman School of Medicine, Philadelphia, PA, USA

**Keywords:** molecular imaging, indocyanine green, near-infrared, margins

## Abstract

Near infrared (NIR) molecular imaging is useful to identify tumor margins during surgery; however, the value of this technology has not been evaluated for tumors that have been pre-treated with chemotherapy. We hypothesized that NIR molecular imaging could locate mediastinal tumor margins in a murine model after neoadjuvant chemotherapy. Flank thymomas were established on mice. Two separate experiments were performed for tumor margin detection. The first experiment compared (i) surgery and (ii) surgery + NIR imaging. The second experiment compared (iii) preoperative chemotherapy + surgery, and (iv) preoperative chemotherapy + surgery + NIR imaging. NIR imaging occurred following systemic injection of indocyanine green. Margins were assessed for residual tumor cells by pathology. NIR imaging was superior at detecting retained tumor cells during surgery compared to standard techniques (surgery alone vs. surgery + NIR imaging, 20% vs. 80%, respectively). Following chemotherapy, the sensitivity of NIR imaging of tumor margins was not significantly altered. The mean *in vivo* tumor-to-background fluorescence ratio was similar in the treatment-naïve and chemotherapy groups ((*p* = 0.899): 3.79 ± 0.69 (IQR 3.29 - 4.25) vs. 3.79 ± 0.52 (IQR 3.40 – 4.03)). We conclude that chemotherapy does not affect tumor fluorescence or identification of retained cancer cells at margins.

## INTRODUCTION

Mediastinal masses comprise a diverse group of benign and malignant tumor types such as thymomas, thymic carcinomas, germ cell tumors and neurogenic tumors [1]. The most important prognostic indicator for mediastinal thymomas and thymic carcinomas is a complete surgical resection [2–4]. In situations such as locally advanced disease, preoperative chemotherapy is frequently used to reduce tumor burden [5–8]. However, pre-treatment of mediastinal tumors can result in local inflammatory responses and scarring that blurs tumor margins. As a consequence this increases the complexity of the operation, risks an incomplete resection and can result in a local recurrence.

Molecular imaging is an emerging technology to improve surgical resections by identifying small tumors, delineating tumor margins, and localizing lymph nodes that may contain metastatic cancer cells during surgery. This technique is based on fluorescent molecular agents and imaging devices that improve tumor visualization. Clinically, intraoperative molecular imaging with a near-infrared (NIR) fluorescent contrast agent, indocyanine green (ICG), has been used for colorectal cancer, lung cancer and sentinel lymph node mapping [9–14].

Our laboratory has been investigating whether this technology is applicable to mediastinal tumors. In animal studies, we have found that ICG provides excellent contrast of mediastinal tumors compared to surrounding structures and tissues. However, the value of this technology has not been tested in a common clinical scenario that would be very valuable: patients who undergo resection following neoadjuvant chemotherapy. Studies have shown that one of the major limitations of ICG is that it has non-specific uptake in inflammatory tissues which limits its use for diagnostic purposes [15]. It has been shown that preoperative chemotherapy increases local inflammation of solid tumors [16, 17]. Specifically, Weissferdt et al showed increased tumor necrosis, cystic changes, hemorrhage and calcification in thymomas treated with preoperative chemotherapy [18]. Thus, it is not evident if neoadjuvant chemotherapy would reduce the value of NIR imaging in delineating tumor margins.

We postulated that the peritumoral inflammatory response following chemotherapy would decrease tumor fluorescence and/or result in inaccurate margin assessment. Thus, in this study, we examined the effect of standard-of-care cisplatin on the sensitivity of intraoperative NIR imaging for detecting mediastinal flank tumor margins.

## RESULTS

### NIR imaging improves detection of murine thymoma tumor margins

**Figure 1:**
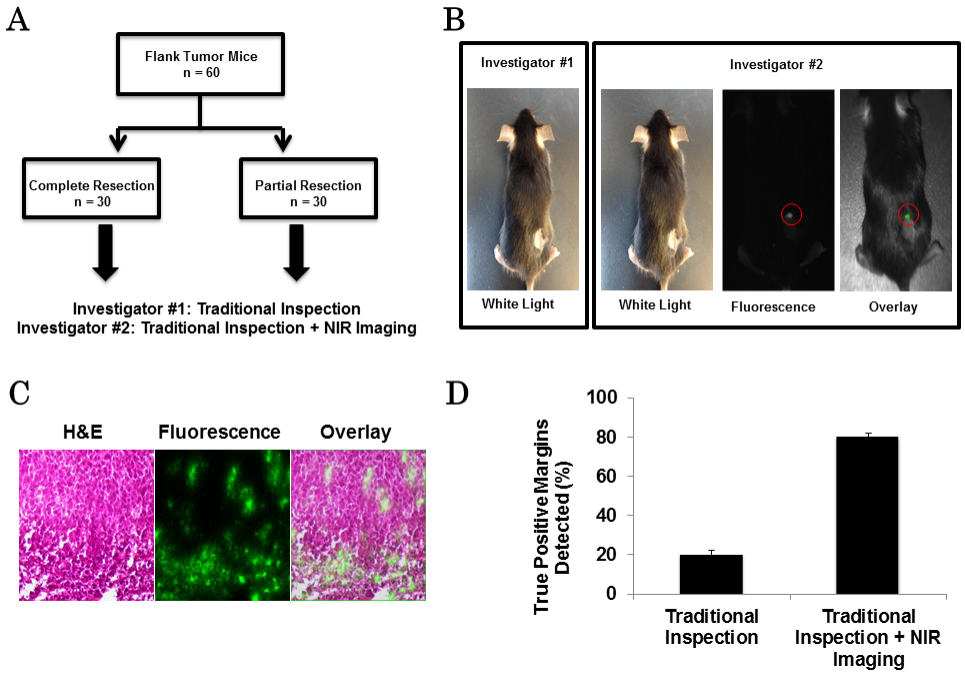
**A.** Schematic of tumor margin assessment model. **B.** Example images from traditional inspection (investigator #1) vs. traditional inspection + NIR imaging (investigator #2) for the detection of positive margins in a 4T1 flank tumor bearing mouse. Circle: positive margin detected by NIR imaging. **C.** H&E, fluorescence microscopy and overlay of EL4 flank tumor after resection. **D.** Sensitivity of positive margin detection of investigator #1 and investigator #2.

In order to determine if NIR imaging could detect residual cancer cells following tumor resection, EL4 (*n* = 30) and EG7 (*n* = 30) flank tumors were established. Once the tumors reached 500 mm^3^, animals underwent surgery and were randomized to complete resection (*n* = 30) versus intentional partial resection with positive surgical margins (*n* = 30). Two independent investigators were asked to inspect the wound bed to locate residual disease (Figure 1a). Investigator #1, using his hands and eyes, identified 9 mice with suspicious surgical margins that may contain tumors cells. Investigator #2 used NIR imaging, and he identified 24 mice with suspicious positive surgical margins (Figure 1b). After both investigators independently assessed the wound, all surgical margins including the suspicious areas were photo-documented, excised and placed in formalin for pathological analysis (Figure 1c).

The first investigator correctly identified 6 positive margins, but falsely selected 3 margins that did not contain cancer cells. The second investigator using NIR imaging correctly located 24 positive margins and did not identify any false positives.

All experiments were repeated. Overall, fluorescent imaging had a sensitivity of 80% and specificity of 100% while visual inspection and manual palpation had a sensitivity of 20% and specificity of 90% (Figure 1d).

### Tumor fluorescence persists after chemotherapy

**Figure 2:**
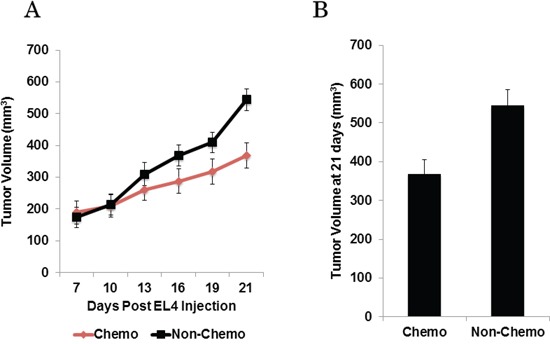
**A.** Murine flank tumor volume plotted over time throughout chemotherapy or control treatment. **B.** Mice receiving chemotherapy have significantly smaller flank tumors at treatment end.

Next, in order to study the effect of chemotherapy on tumor fluorescence, additional EL4 and EG7 flank tumors (*n* = 60) were established. One week following tumor cell injection, all tumors were easily identifiable but small in size (< 200 mm^3^). Half of the mice were randomized to the intraperitoneal cisplatin whereas the other animals received saline injections over 3 weeks. Tumor volume was measured twice weekly during treatment (Figure 2a). Three weeks after initiating chemotherapy (and prior to any tumor resection) all animals underwent molecular imaging and were euthanized for correlative studies.

By four weeks following initial tumor cell injection, the mean tumor volume of the animals that had undergone chemotherapy was 368 ± 37 mm^3^, while the mean tumor volume of the non-chemotherapy group was 544 ± 41 mm^3^ (Figure 2b). The tumors of animals that received chemotherapy were significantly smaller than the non-chemotherapy group (*p* < 0.0001).

**Figure 3:**
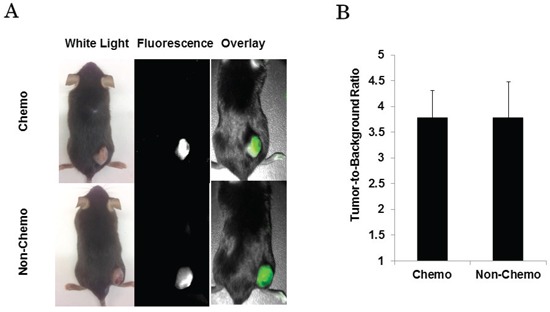
**A.** Mice imaged with NIR camera prior to undergoing tumor resection. **B.** Regardless of treatment status, mice have similar mean TBR when measured with NIR imaging device.

The investigators subjectively detected fluorescence in all flank tumors regardless of chemotherapy status. Mice that received cisplatin (Figure 3a) had a mean tumor fluorescence of 3.88 (IQR 3.77 - 4.10), a mean background fluorescence of 1.03 (IQR 0.95–1.12), and a mean TBR of 3.79 ± 0.52. Control mice (Figure 3b) had a mean tumor fluorescence of 3.76 (IQR 3.46–3.91), a mean background fluorescence of 1.01 (IQR 0.94–1.12), and a mean TBR of 3.79 ± 0.69. There was no significant difference between the mean TBR between the mice that received chemotherapy versus control group (*p* = 0.99).

In summary, this suggests that tumors that had a treatment response to chemotherapy were equally fluorescent to non-treated tumors. In addition, tumor size did not correlate with tumor fluorescence.

### Quantification of tumor fluorescence by spectroscopy

After quantifying ICG fluorescence using a NIR camera as described above, in order to rigorously confirm tumor fluorescence, we measured signal intensity from these same murine thymomas (cisplatin and saline treated mice) using a hand-held NIR fluorescence spectroscopy device. First, we measured the background fluorescence signal from skin distant from the flank tumors. Next, we measured the NIR signal intensity (again at 820 nm) from the middle of each flank tumor and from three distinct tumor locations approximately 2 mm from the center of the flank tumor. A total of four readings from both skin and tumor were taken in each animal and averaged to find the mean fluorescence intensity.

The measurements varied widely in all cases. The mean background fluorescence from skin from all mice was 842 ± 316, whereas the mean tumor fluorescence from all mice was 57478 ± 1022. The mean TBR of the tumor center to the surrounding skin was 68.3 (IQR 57.2 – 80.6).

**Figure 4:**
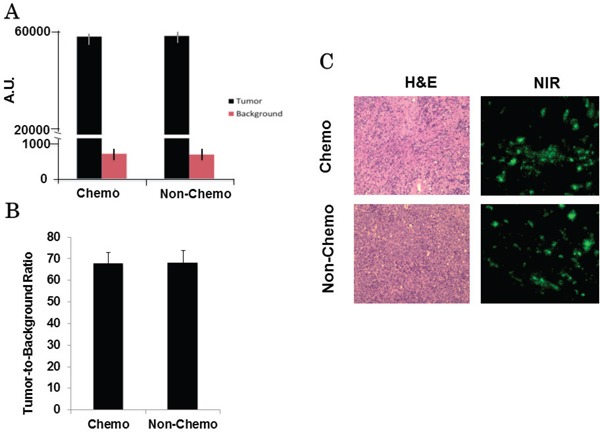
**A.** A hand-held spectrometer confirms similar tumor and background fluorescence between both chemotherapy and control group mice. **B.** Calculated mean spectroscopic TBRs are similar between both groups. **C.** Example of H&E (20x) and fluorescence microscopy (20x) for a chemotherapy-treated and control tumor.

We then compared the NIR fluorescence intensities between the control and chemotherapy-treated animals. The mean cutaneous background fluorescence from control animals was 819 ± 198, the mean tumor fluorescence from these mice was 56222 ± 783, and mean TBR was 68.7 (IQR 55.4 – 83.1). The mean background fluorescence from chemotherapy-treated animals was 859 ± 212, the mean tumor fluorescence was 58113 ± 754, and mean TBR was 67.7 (IQR 58.3 – 82.2) (Figure 4a). The mean spectroscopic TBR between both groups was not significantly different (*p* = 0.78) (Figure 4b). These findings supported our previous results that neither size nor chemotherapy treatment status affected the intensity of NIR fluorescence.

Following confirmation of macroscopic fluorescence using both NIR imaging and spectroscopy, tumors were prepared for frozen section as described. Tumor specimens underwent fluorescence microscopy and H&E for evidence of chemotherapeutic changes. Microscopic mean TBR of the chemotherapy-treated tumors was 6.7 and mean TBR of control tumors was 6.4. Despite the similarity in fluorescence between the two groups, tumors treated with chemotherapy were found to have a higher degree of tumor necrosis, fibrosis and lymphocytic reaction (Figure 4c).

### Pre-operative chemotherapy does not effect detection of thymoma flank tumor margins

**Figure 5:**
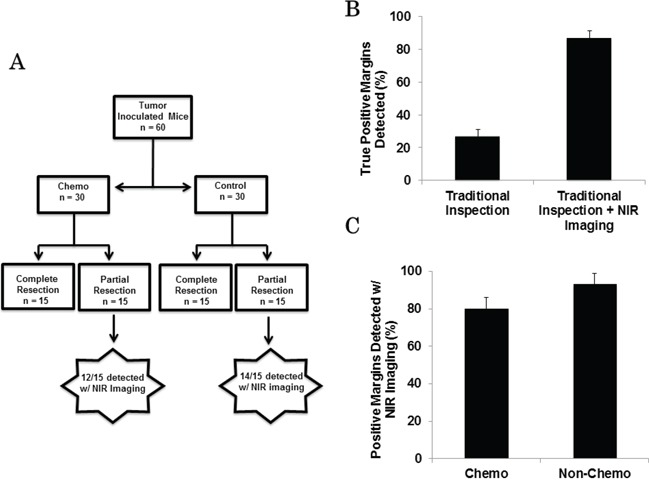
**A.** Schematic representing margin assessment study comparing detection of positive tumor margins in both chemotherapy and control group animals. **B.** Sensitivity of positive margin detection of investigator #1 and investigator #2. **C.** Rate of margin detection between mice that received chemotherapy and those that did not is similar.

Next, we studied the effect of pretreatment chemotherapy on assessing surgical margins. In order to do this, we used the same experimental set-up described above. Animals were randomized to either 3 weeks of cisplatin or control treatment. We then repeated the margin experiment, assuring that half of the mice from each treatment group (chemotherapy vs. control) were randomized to either complete resection versus partial resection with intentional positive surgical margins. Similar to the first experiment, a total of 30 mice underwent complete tumor excision while the remaining 30 mice underwent partial tumor excision (Figure 5a).

Again, two independent investigators were asked to inspect the wound bed to locate residual margin following tumor excision. The first investigator using visual inspection and manual palpation identified 10 mice with suspicious positive surgical margins. The second investigator, who used NIR imaging in addition to traditional means, identified 26 mice with suspicious positive tumor margins. Eight of the 10 margins detected by the first investigator and all 26 margins located with NIR imaging were confirmed to be tumor on histopathology (Figure 5b).

Of these 26 animals with confirmed positive margins that were noted by the second investigator using the NIR camera, 12 had received preoperative cisplatin and the other 14 animals had received the control treatment. NIR imaging was highly successful in detecting positive margins in both the chemotherapy treated animals (80%) and the non-chemotherapy group (93%) (Figure 5c).

## DISCUSSION

NIR intraoperative molecular imaging is a rapidly emerging tool for detecting tumor cells, metastases and lymph nodes with cancer cells [9–12]. Additionally, this technology has shown value for identifying tumor cells in the surgical wound following cancer surgery. For example, preliminary results from our laboratory have shown success in predicting tumor margins following resections of mediastinal tumors and extremity sarcomas. However, to our knowledge, the impact of preoperative chemotherapy on intraoperative molecular imaging of tumor margins and surgical margins has not been rigorously assessed.

This study had two major findings. First, we found that using NIR imaging of mediastinal tumor margins was superior to traditional methods alone such as manual palpation and visual examination. We were able to identify residual tumors cells three-fold more frequently in those animals that had surgery with NIR imaging compared to conventional surgery. Previously, our lab has shown superior tumor margin detection using NIR imaging with other tumor models [19]. Here, we confirm this finding in a murine thymoma model. Overall, the addition of NIR imaging added little additional time to the inspection of the wound bed. In fact, the argument could be made that had the second investigator used the NIR camera prior to traditional inspection of the wound bed, the overall time to detect positive tumor margins may have been reduced when compared to traditional inspection alone, as the residual fluorescence is immediately evident with NIR imaging.

Second, contrary to our initial hypothesis, we discovered that murine thymomas had similar NIR fluorescence irrespective of preoperative chemotherapy status. We assessed tumor fluorescence using NIR spectroscopy for precise quantification of the tumor-to-background signal, and this rigorous analysis showed no difference in tissue fluorescence. These results show that a decrease in tumor size did not affect the intensity of tumor fluorescence. This supports our prior human data that shows tumor size does not seem to affect tumor fluorescence [11]. We postulate the lack of correlation between tumor size and fluorescence may be due to two reasons. First, our NIR imaging and spectroscopic devices may lack sufficient resolution to identify subtle differences in fluorescence. Second, ICG is thought to accumulate in tumors by the enhanced permeability and retention (EPR) effect. The EPR effect is related to high intratumoral pressures, widely fenestrated neovasculature and lack of lymphatics which selectively allows nanoparticle-sized particles to accumulate in tumors [20, 21]. Although our pre-treated murine thymomas were smaller in size, it is possible that the “leaky” intratumoral vasculature was unaffected or even more disrupted. In order to more fully understand why chemotherapy or tumor size did not affect fluorescence, further studies should be conducted to elucidate the mechanism and binding of ICG.

Not all of the tumor deposits left by the surgeon in our murine model were discovered with fluorescence imaging, and there are several reasons why this may have occurred. It is possible that the tumor piece left behind was so small that the camera lacked sufficient resolution to detect a small area of fluorescence. Additionally, with the manipulation of the tumor, the tracer may have been manually removed, especially since ICG is not receptor bound. Lastly, it is also possible that upon hiding the residual tumor, it was placed too deep in the wound bed so that the camera/tracer combination lacked sufficient depth of penetration.

There are several limitations to this study. First, the origin of the EL4 and EG7 cell lines is controversial. Some groups have speculated that these cell lines are lymphomas and not thymomas [22–24]. Second, human thymomas are rarely treated with preoperative chemotherapy. A preferable model would be a murine thymic carcinoma cell line; however, to our knowledge, this does not exist. Third, due to the short course of a mouse life span, we could only give an abbreviated course of preoperative chemotherapy. Additionally, a longer treatment course would have created an unacceptable degree of tumor burden in the control especially in non-treated animals. It would be worthwhile to repeat the experiment with a slow growing cell line in order to appreciate the effects of a longer course of chemotherapy.

These results are encouraging because they suggest that patients who have undergone neoadjuvant chemotherapy are reasonable candidates for NIR intraoperative molecular imaging. Continued research to evaluate other tumor types and the impact of preoperative radiation are ongoing. This study is the basis for an ongoing clinical trial of NIR imaging of mediastinal tumors.

## MATERIALS AND METHODS

### Cell lines

The murine malignant thymoma cell line, EL4, was established from thymoma induced in a C57BL/6 mouse and has been previously described in detail [25]. EG7-OVA is a derivative of the EL4 cell line that was stably transfected with OVA complementary DNA. All cells were cultured and maintained in RPMI-1640 with 2 mM L-glutamine, 10% FBS, plus 1.5ul of 2-mercaptoethanol / 500 ml, and were passed every 2–3 days to avoid overgrowth. The cell lines were regularly tested and maintained negative for *Mycoplasma spp*.

### Reagents

Indocyanine green (ICG) (Akorn, Lake Forest, IL and Pulsion, Feldkirchen, Germany) is a water-soluble anionic, amphiphilic NIR fluorophore with an excitation wavelength of 790nm and an emission wavelength of 830nm and a molecular weight of 774.9 kDA. Animals received 5mg/kg of ICG via tail-vein injection 24 hours prior to NIR imaging.

### Near-Infrared Imaging

Macroscopic surgical fluorescent imaging was performed using the FloCam (BioVision, Exeter, PA). This imaging device was developed in our laboratory and is capable of dual imaging with white light and NIR. The FloCam parameters are easily kept consistent across each animal as the exposure time (40 msec) and focal length (20 cm) were maintained at one value. For this reason, we were able to measure accurate and consistent fluorescence for each animal. Positive and negative controls were used for all images.

We used region-of-interest software and HeatMap plugin within ImageJ (http://rsb.info.nih.gov/ij/; public domain free software developed by National Institutes of Health) in order to quantitate tissue fluorescence. Background readings were taken from normal cutaneous tissue in order to generate a tumor-to-background ratio (TBR). All readings were done in quadruplicate.

### Spectroscopy

NIR spectroscopy was performed using a hand-held device (Spectropen, InPhotonics, Norwood MA). This device measured NIR fluorescence of the flank tumors of mice that had undergone either chemotherapy or control treatment regimens. The Spectropen is a cylindrical stainless steel device comprised of illumination and detector fibers coupled via an FC connector to a spectrometer. The spectrometer utilizes a NIR diode laser (λex 785 nm) coupled to a head unit for light excitation and collection. During use, the device was held as close as possible to the tissue to be measured without making contact. We took four spectroscopic readings of the tumor as well as the adjacent normal cutaneous tissue as background. The illumination intensity and detector integration time (0.1 sec) were held constant across all measurements, which allowed for consistency in the measured fluorescence detection [26].

### Murine flank tumor model for mediastinal margin assessment

All experiments were approved by the University of Pennsylvania Animal Use Committee and are in compliance with the Guide for the Care and Use of Laboratory Animals. Sixty 6-week-old mice (C57BL/6) were injected subcutaneously in the flank with 1.0 × 10^6^ EL4 or EG7 cells suspended in 100μL PBS. Tumor volume was periodically calculated using the formula (3.14 x long-axis x short-axis^2^)/6. When tumors reached ~ 500 mm^3^, all mice were injected 24 hours prior to surgery via tail vein with 5 mg/kg of ICG dissolved in deionized water. One day after injection, mice were anesthetized with intramuscular ketamine (80 mg/kg) and xylazine (10 mg/kg), shaved, and then sterilized prior to *in vivo* imaging.

We then used these mice in order to assess the utility of NIR intraoperative imaging in the detection of positive surgical margins. Following initial *in vivo* imaging as described above, a 1-to-2 cm incision was made adjacent to the tumor and the tumor was removed using standard blunt and sharp dissection techniques. The surgeon intentionally left approximately 5% of tumor hidden within the fur and/or soft tissue of the surgical margin in 30 of the 60 mice as previously described [27]. Next, two blinded independent investigators inspected the surgical field all mice in an attempt to discover retained tumor. The first investigator, who was blinded to the results of the tumor excision, was allowed to use his hands and eyes only to search for retained tumor in the tumor bed. The second investigator, who was blinded to both the surgeon and the first investigator, then used these traditional methods (visual inspection and palpation) as well as NIR fluorescence imaging to evaluate the tumor bed for residual tumor. Suspicion for residual tumor was defined as any tissue that appeared to be additional tumor based on inspection and palpation or an area of retained tissue bed fluorescence with TBR ≥ 1.5. After both investigators were finished, all surgical beds including each true positive wound bed (determined by the initial surgeon) as well as suspected positive margins chosen by each investigator were biopsied for H&E. Presence of residual tumor on wound bed biopsy was the gold standard of a positive surgical margin in these experiments, and in all cases, negative margins were confirmed with H&E of wound bed biopsies.

### Chemotherapy

Additional thymoma flank tumors (*N* = 60) were established in order to assess the effect of chemotherapy on intraoperative imaging of mediastinal tumors and tumor margins. Chemotherapy was prepared by dissolving 8 mg of powdered cisplatin (EMD Millipore, Billerica, MA) into 400 uL of dimethylformamide, which was diluted to 4mg/kg in phosphate buffered saline. 30 mice were randomized to either the chemotherapy or control groups. Starting one week following tumor cell injection, the solution was given intraperitoneally using a 25 gauge needle twice weekly for 3 weeks. The equivalent amount of normal saline was administered to the remaining 30 mice using the identical biweekly injection schedule. Tumor volume was calculated using the formula (3.14 x long-axis x short-axis^2^)/6 twice weekly to evaluate tumor growth. Following the three weeks of treatment, all mice were injected 24 hours prior to surgery via tail vein with 5 mg/kg of ICG dissolved in deionized water. The flank tumors were inspected and imaged *in vivo* using both a NIR imaging device and hand-held spectrometer. The mean values were then used to calculate the mean tumor-to-background ratio (TBR) for each animal.

An identical margin assessment study as described previously was then carried out to assess the effect of chemotherapy on tumor margin detection. In this experiment, half of the animals from both the chemotherapy and control groups underwent partial resections. The remaining animals had complete resection of their flank tumors. All experiments were repeated in triplicate.

### Histopathology and fluorescence microscopy

Histopathology was used to evaluate all resected tissues. Samples were fixed in 10% formalin, embedded in paraffin, sectioned, and evaluated by a board certified veterinary pathologist. Fluorescence microscopy was utilized to verify the accumulation of ICG within resected tissues in several samples. 5 μm-thick sections from tissue biopsies obtained during the surgery were mounted with a glycerin-based mounting media and frozen tumor sections were prepared as previously described [28]. The samples were examined using an Olympus^®^ IX51 fluorescent microscope equipped with an indocyanine green specific filter set (Chroma^®^ 49030). Fluorescence images were acquired using a PixeLink^®^ NIR CCD camera (PL-B741EU). Each sample was then subsequently stained with hematoxylin and eosin and re-imaged using white light. Fluorescence images were processed using ImageJ^®^ (http://rsb.info.nih.gov/ij/; public domain software developed by National Institutes of Health). Using this software, TBR was measured for each fluorescence microscopy image. Fluorescence was measured across the entirety of the tumor, whereas background was measured from the surrounding glass slide. The mean TBR of both the chemotherapy-treated and control were then compared.

### Statistics

The sensitivity and specificity for margin detection were calculated for both the first and second investigators involved in the margin assessment study. We then compared the mean TBR (using both the NIR imaging device and spectrometer) between the animals who received chemotherapy and those who did not using a Student's *t*-test. Similarly, the size of the tumors was evaluated for significant difference between the two treatment groups also using a Student's *t*-test. Lastly, the sensitivity of NIR imaging for the detection of positive margins was compared between the mice that received chemotherapy and those who did not. Calculations were performed in Excel (Microsoft^®^) and SAS^®^ software.
